# *WNT3A* rs752107(C > T) Polymorphism Is Associated With an Increased Risk of Essential Hypertension and Related Cardiovascular Diseases

**DOI:** 10.3389/fcvm.2021.675222

**Published:** 2021-07-12

**Authors:** Huan Ren, Jian-Quan Luo, Fan Ouyang, Li Cheng, Xiao-Ping Chen, Hong-Hao Zhou, Wei-Hua Huang, Wei Zhang

**Affiliations:** ^1^Department of Clinical Pharmacology, Xiangya Hospital, Central South University, Changsha, China; ^2^Hunan Key Laboratory of Pharmacogenetics, Institute of Clinical Pharmacology, Central South University, Changsha, China; ^3^Engineering Research Center of Applied Technology of Pharmacogenomics, Ministry of Education, Changsha, China; ^4^National Clinical Research Center for Geriatric Disorders, Changsha, China; ^5^Department of Pharmacy, The Second Xiangya Hospital, Central South University, Changsha, China; ^6^Department of Cardiology, Zhuzhou Central Hospital, The Affiliated Zhuzhou Hospital of Xiangya Medical College of Central South University, Zhuzhou, China

**Keywords:** essential hypertension, heart failure, ischemic stroke, Wnt3a, genetic polymorphisms

## Abstract

Essential Hypertension (EH) results in the burden of cardiovascular disease (CVD) such as Heart Failure (HF) and Ischemic Stroke (IS). A rapidly emerging field involving the role of Wnt/β-catenin signaling pathway in cardiovascular development and dysfunction has recently drawn extensive attention. In the present study, we conducted a genetic association between genomic variants in Wnt/β-catenin signaling pathway and EH, HF, IS. A total of 95 SNPs in 12 Wnt signaling genes (*WNT3A, WNT3, WNT4, DKK1, DKK2, LRP5, LRP6, CTNNB1, APC, FZD1, FRZB, SFRP1*) were genotyped in 1,860 participants (440 patients with EH, 535 patients with HF, 421 patients with IS and 464 normal control subjects) using Sequenom MassArray technology. *WNT3A* rs752107(C > T) was strongly associated with an increased risk of EH, HF and IS. Compared with *WNT3A* rs752107 CC genotype, the CT genotype carriers had a 48% increased risk of EH (OR = 1.48, 95% CI = 1.12–1.96, *P* = 0.006), the TT genotype conferred a 139% increased risk of EH (OR = 2.39, 95% CI = 1.32–4.34, *P* = 0.003). Regarding HF and IS, the risk of HF in the T allele carriers (CT + TT) was nearly increased by 58% (OR = 1.58, 95% CI = 1.22–2.04, *P* = 4.40 × 10^−4^) and the risk of IS was increased by 37% (OR = 1.37, 95% CI = 1.04–1.79, *P* = 0.025). Expression quantitative trait loci (eQTL) analysis indicated that rs752107 C allele corresponded to a significant reduction of *WNT3A* expression. We described a genetic variant of *WNT3A* rs752107 in Wnt/β-catenin signaling strongly associated with the risk of EH, HF and IS for the first time.

## Introduction

Cardiovascular disease (CVD) refers to the pathologies associated with the heart and circulation system, including cerebrovascular, and cardiovascular. CVD is a crisis for human health that plays a vital role on human morbidity and mortality. Substantial evidence suggests that CVD is heritable, and genetic predisposition have long been thought to contribute to the development and progression of this complex disease. With the rapidly evolving of high throughput sequencing, genetic studies have organized large amounts of gene variants across the human genome that are associated with CVD susceptibility (1, 2).

Hypertension is the predominant independent risk factor for CVD. Observational investigations and clinical trials have demonstrated that long periods of uncontrolled hypertension are responsible for the catastrophic events, such as heart attack and stroke (3, 4). Hypertension is caused by the complex interaction of environmental and pathophysiological, as well as genetic predisposition. The evidence for a genetic basis of hypertension provides valuable insights into blood pressure regulation. Based on genome wide association studies (GWAS), over 100 single nucleotide polymorphisms (SNPs) associated with blood pressure phenotypes have been identified (5). Although remarkable progress in genetic or genomic characterization of hypertension and CVD phenotypes has been made, the known genetic markers are still far from explaining the susceptibility or prognosis of these diseases. There is an urge for development of novel markers to enable better prevent, diagnosis, prognosis, and efficient treatment.

Wnt proteins, one family of growth factors, are secreted glycoproteins important for cellular proliferation and differentiation, tissue morphogenesis, and tissue homeostasis (6–8). Wnt signaling pathway is critically important for development of cardiac, vascular, and endothelial cell specification. Evidences showed that both loss- and gain-of-function of Wnt signaling pathway may cause marked alterations of angiogenesis, cardiovascular development and endothelial cell specification (9–11). Normally, few Wnt signaling activity is present in the cardiovascular system of healthy adults. Excessive activation of Wnt signaling in the cardiovascular system may contribute to cardiovascular disease (12). Indisputably, dyfunction of Wnt signaling is factitive to multiple pathologies of heart and blood vessels. Nevertheless, little evidence has described the genetic association between polymorphisms in Wnt signaling pathway and EH, HF, IS.

Based on the identity of Wnt ligands or receptors, Wnt signaling pathway can be subdivided into canonical and non-canonical. The canonical Wnt signaling pathway involves the elevated levels of β-catenin in cells. β-catenin functions as intracellular signaling protein by interacting with transmembrane proteins of the cadherin family. It has been suggested that low-density lipoprotein-related receptor 6(LRP6), a key receptor of Wnt/β-catenin signaling, is involved in the development of cardio-cerebrevascular diseases. Mani et al. found a missense mutation (R611C) in LRP6, which substitutes cysteine for arginine at highly conserved residue of an EGF-like domain and impairs Wnt signaling *in vitro*, contributes to the metabolic syndrome including early coronary artery disease, hyperlipidemia, hypertension, and diabetes (13). Sarzani et al. indicated that LRP6 I1062V (rs2302685) was associated with carotid artery atherosclerosis (CAA) through retrospective analysis of 334 secondary hypertensive patients and the expression of LRP6 was significantly decreased in atherosclerotic plaque of patients with hypertension (14). Therefore, we hypothesized that the Wnt/β-catenin signaling genes were mainly associated with hypertension and related CVDs. And the aim of this study was to explore the role of the mutated Wnt/β-catenin signaling pathway components on genetic predisposition of EH, HF, and IS.

In the present study, we conducted case–control studies and screened a total of 95 potentially functional variants within 12 Wnt/β-catenin signaling genes (*WNT3A, WNT3, WNT4, DKK1, DKK2, LRP5, LRP6, CTNNB1, APC, FZD1, FRZB, SFRP1*). At first, we confirmed a genetic association of *WNT3A* rs752107 with EH susceptibility in exploration cohort (199 patients with EH and 135 healthy subjects) and enlarged validation cohort (440 patients with EH and 464 healthy subjects). Then, *WNT3A* rs752107 was analyzed in HF cohort (535 patients) and IS cohort (421 patients). We determined the novel association between genomic variants in Wnt/β-catenin signaling pathway and EH and related CVDs.

## Materials and Methods

### Participants

All participants were recruited from the Xiangya Hospital (Changsha, China). Patients with EH and HF were recruited from Department of Cardiology, Xiangya Hospital, Central South University. According to the universal diagnostic criteria, hypertension was defined as blood pressure above 140/90 mmHg or taking antihypertensive drugs. Here we excluded secondary hypertension patients. Patients with IS were recruited from Department of Neurology, Xiangya Hospital, Central South University. Included patients with IS were ruled out hemorrhagic and cardioembolic stroke. Control group selected from visitors at Physical Examination Center for a routine checkup. Exclusion criteria for health control were hypertension, coronary heart disease, ischemic cardiomyopathy, diabetes, HF, and IS. Medical history, demographic data and anthropometric data were obtained by questionnaire. All subjects gave their informed consent for inclusion before they participated in the study. The study was conducted in accordance with the Declaration of Helsinki, and the protocol was approved by the Medical Ethics Committee of Xiangya Hospital, Central South University.

### SNP Selection

Candidate genes were chosen from Wnt/β-catenin genetic pathways. The selection of SNPs was based on public information available in dbSNP and Hapmap by Haploview 4.0 with a *r*^2^ threshold of 0.8. The criteria for the SNPs were as below: minor allele frequency >5% in Chinese, and with potential functions. A total of 95 SNPs in 12 Wnt/β-catenin signaling genes (*WNT3A, WNT3, WNT4, DKK1, DKK2, LRP5, LRP6, CTNNB1, APC, FZD1, FRZB, SFRP1*) were selected. The genes, SNP IDs, and their information locations are exhibited in Supplementary Table 1.

### SNP Genotyping

Genomic DNA was extracted from 3 ml of venous blood samples using Blood DNA Maxi Kit (OMEGA, D3392) according to the manufacturer's protocol.

In the exploration cohort, genotyping was performed using MassARRAY high-throughput DNA analysis with matrix-assisted laser desorption/ionization time-of-flight mass spectrometry (Sequenom, Inc., San Diego, CA, USA). 10% of samples were randomly selected for genotyping quality control. Subsequently, SNPs were genotyped using iPLEX Gold technology (Sequenom) followed by an automated data analysis with the TYPER® RT software version 4.0. In the enlarged validation cohort and HF, IS cohort, *WNT3A* rs752107 was genotyped by DNA sequencing after polymerase chain reaction (PCR).

### Expression Quantitative Trait Loci (eQTL) Analysis

To explore the effects of genetic variation in multiple human reference tissues, we used the Genotype-Tissue Expression (GTEx) project database.

### Statistical Analysis and Graphical Presentations

The statistical analyses of collected data and clinical results was performed with SPSS version 20.0 and PLINK 1.07 software. Quantitative data were expressed as mean value and standard deviation (SD). Genotype and allele frequencies were compared by direct counting. Categorical data between groups and deviations from Hardy–Weinberg equilibrium were tested by Chi-square test. According to distribution of our data, *t*-test or Mann–Whitney *U* Tests were used for between-groups' comparisons. The association between CVD and Wnt/β-catenin signaling genes polymorphism was expressed as the odds ratios (ORs) and 95% confidence intervals (CIs). The Bonferroni correction was used to control for multiple testing. Schematic diagram of Wnt/β-catenin signaling pathway was accomplished by ScienceSlides 6.0.

## Results

### Baseline Characteristics

The exploration cohort included 199 EH patients and 135 controls. The enlarged validation cohort, which absorbed samples from exploration cohort, included 440 patients with EH and 464 healthy subjects at all. Baseline characteristics of enlarged EH cohort and controls, as well as HF and IS cohorts, are shown in Supplementary Table 2.

### Genotype Distribution in EH Cases and Controls

9 SNPs were removed from further analysis for any of the following motives: Monomorphism, call rate below 95%, Minor Allele Frequency (MAF) <5%, and deviation from hardy–weinberg equilibrium. Eighty six valid SNPs were included in the statistics (Supplementary Table 3).

In the exploration cohort, the analysis results showed that seven of candidate SNPs (*WNT3A* rs752107, rs1636195, rs3121310, rs1745423, *FRZB* rs4666865, *SFRP1* rs12914, *DKK2* rs419764) were significantly associated with EH (Table 1). However, after correction for multiple testing, only *WNT3A* rs752107(C > T) polymorphism remained significant correlation. The frequency of T allele in patients with EH was significantly increased than the controls (29.6 vs. 16.8%; *P* = 2.2 × 10^−4^). Therefore, further exploration was performed to confirm the genetic association of *WNT3A* rs752107 with EH susceptibility in an enlarged validation cohort.

**Table 1:** SNPs associated with EH in the exploration cohort.

**SNP**	**Gene**	**F_A**	**F_U**	**CHISQ**	***P***	**OR**	**L95**	**U95**
rs1636195	*WNT3A*	0.097	0.034	9.516	0.002	3.08	1.46	6.52
rs1745423	*WNT3A*	0.486	0.399	4.823	0.028	1.43	1.04	1.97
rs3121310	*WNT3A*	0.420	0.330	5.399	0.020	1.47	1.06	2.04
rs752107	*WNT3A*	0.296	0.168	13.640	0.000	2.08	1.40	3.08
rs4666865	*FRZB*	0.207	0.130	6.621	0.010	1.76	1.14	2.70
rs419764	*DKK2*	0.187	0.263	5.428	0.020	0.64	0.44	0.93
rs12914	*SFRP1*	0.058	0.113	6.266	0.012	0.49	0.27	0.86

*SNP, single nucleotide polymorphism; EH: essential hypertension; F_A, frequency in case group; F_U, frequency in control group; OR, Odds Ratio; L95, 95% confidence lower limit; U95, 95% confidence upper limit*.

In the enlarged validation cohort, as expected, the frequency of T allele in patients with hypertension was significantly increased than the controls (26.6 vs. 19.2%, *P* = 1.74 × 10^−4^). Compared with *WNT3A* rs752107 CC genotype, the CT genotype carriers had a 48% increased risk of EH (OR = 1.48, 95% CI = 1.12–1.96, *P* = 0.006), the TT genotype conferred a 139% increased risk of EH (OR = 2.39, 95% CI = 1.32–4.34, *P* = 0.003) (Table 2).

**Table 2:** Association of *WNT3A* rs752107 variant with EH, HF, and IS.

**Genotype/Allele**	**Control**	**EH**	**OR(95% CI)**	***P***	**HF**	**OR(95% CI)**	***P***	**IS**	**OR(95% CI)**	***P***
CC	304	240	Reference	0.001^*^	292	Reference	0.002^*^	245	Reference	0.076^*^
CT	142	166	1.48 (1.12, 1.96)	0.006	215	1.58 (1.21, 2.06)	0.001	154	1.35 (1.01, 1.79)	0.040
TT	18	34	2.39 (1.32, 4.34)	0.003	28	1.62 (0.88, 2.99)	0.121	22	1.52 (0.80, 2.89)	0.203
CT+TT	160	200	1.58 (1.21, 2.07)	0.001	243	1.58 (1.22, 2.04)	4.40 × 10^−4^	176	1.37 (1.04, 1.79)	0.025
C	750	646	Reference		799	Reference		644	Reference	
T	178	234	1.53 (1.22, 1.91)	1.74 × 10^−4^	271	1.43 (1.15, 1.77)	0.001	198	1.30 (1.03, 1.63)	0.025

* *Means global P-value; OR, odds ratio; 95% CI, 95% confidence index; EH, essential hypertension; HF, heart failure; IS, ischemic stroke*.

### Association Between *WNT3A* rs752107 and HF, IS Susceptibility

Hypertension is the most important contributing cause to other CVDs. Evidences show that long periods of uncontrolled high blood pressure are responsible for the consequent catastrophic events, such as HF and IS. Meanwhile, dyfunction of Wnt Signaling is considered to be a causative factor to vascular and cardiac disease. Reactivation of Wnt signaling is observed in multiple pathologies of heart and blood vessels (12). Therefore, we then explored the association of *WNT3A* rs752107 with the risk of HF and IS. Baseline Characteristics of IS and HF patients are shown in Supplementary Table 2. The control cohort for IS and HF is in common with controls for EH in the enlarged validation cohort. Interestingly, the results suggested strong links between *WNT3A* rs752107 and the risk of IS and HF in line with expectation (Table 2). The frequency of T allele in patients with HF and IS was significantly higher than the controls (25.3 vs. 19.2%, *P* = 0.001; 23.5 vs. 19.2%, *P* = 0.025; respectively). Compared with *WNT3A* rs752107 CC genotype, the risk of HF in the CT genotype carriers was nearly increased by 60% (OR = 1.58, 95% CI = 1.21–2.06, *P* = 0.001) and the risk of IS was increased by 35% (OR = 1.35, 95% CI = 1.01–1.79, *P* = 0.04).

### eQTL Analysis

**Figure 1:**
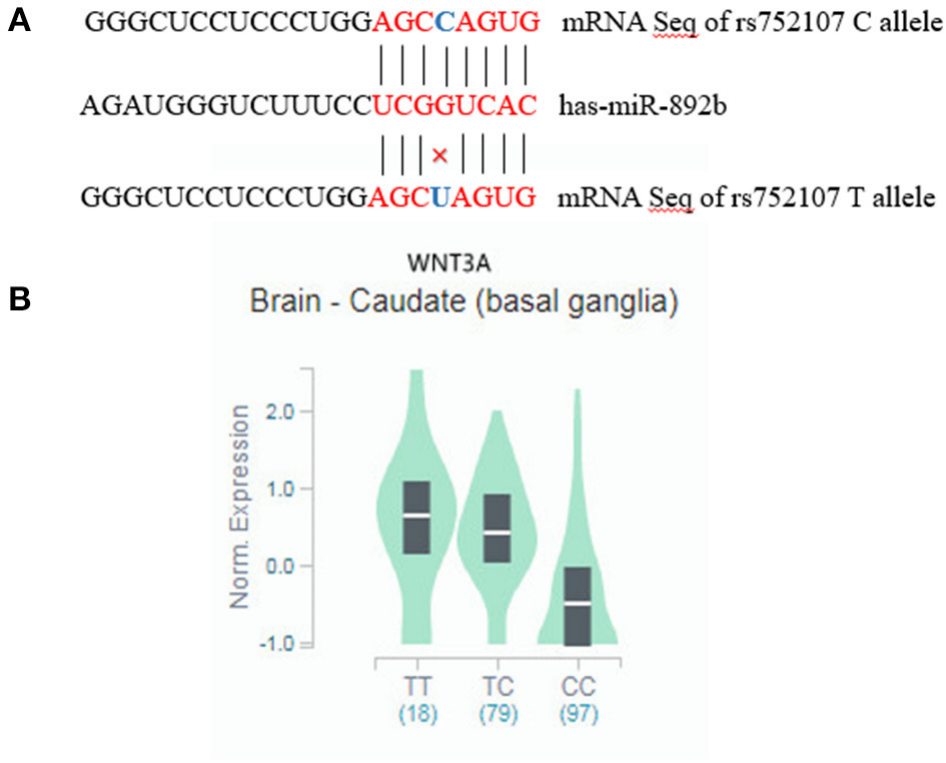
**(A)** mRNA alignment analysis of has-miR-892b with rs752107. **(B)** *WNT3A* mRNA expression level among the different genotypes of rs752107 in brain.

The rs752107 polymorphism is located in 3'untranslated regions (3'-UTRs) involved in gene expression control by binding to MicroRNAs (miRNAs) (15), which may influence transcription of proximal genes. Mirsnpscore (http://www.bigr.medisin.ntnu.no/mirsnpscore) and MirSNP databases (http://cmbi.bjmu.edu.cn/mirsnp) were used to predict the effect of rs752107 polymorphism on gene regulation. The presence of T allele is predicted to break the binding site for has-miR-892b which may lead to an increased level of secreted Wnt3a ligand (Figure 1A). And C allele is predicted to a stronger miRNA-mRNA interaction which may result in a decreased level of *WNT3A* gene expression (*p* = 2.5e−12, Figure 1B).

eQTL analysis was used to evaluate the association of rs752107 with gene expression. Using the GTEx database, rs752107 was found to be associated with differential expression of *WNT3A* in 12 human tissues (Table 3). And C allele corresponded to a significant reduction of *WNT3A* expression in brain.

**Table 3:** rs752107 variant and *WNT3A* expression in 12 human tissues.

**Tissue**	**Samples**	**Beta**	***P***
Skin-not sun exposed	517	0.00135	1
Lung	51	0.0121	0.7
Vagina	141	0.0370	0.5
Testis	322	−0.0570	0.5
Cells-EBV-transformed lymphocytes	147	0.0702	0.5
Prostate	221	−0.0400	0.4
Skin-sun exposed	605	0.0289	0.3
Kidney-cortex	73	0.171	0.2
Minor salivary glad	144	0.0956	0.2
Breast-mammary tissue	396	0.0641	0.2
Esophagus-mucosa	497	0.0711	0.1
Brain-caudate (basal ganglia)	194	−0.638	2.5e−12

## Discussion

**Figure 2:**
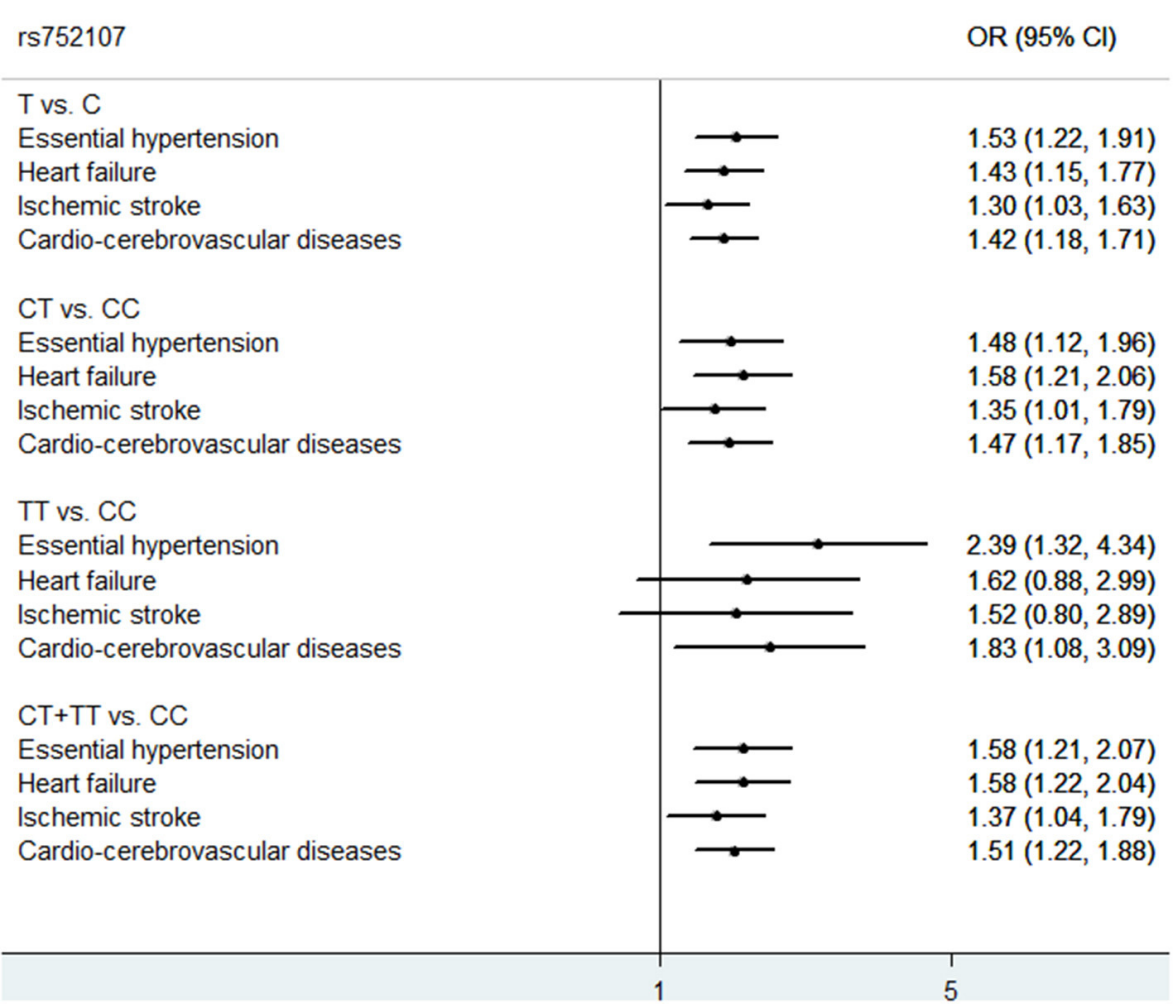
Forest plot for genetic association of *WNT3A* rs752107 variant with EH, HF, and IS.

Hypertension is associated with an increased risk of CVD and is the predominant risk factor for all-cause morbidity and mortality globally. Genetic factors are proved to get involve in the development and progression of hypertension and related CVDs. Here, we validated a novel genomic variant of *WNT3A* rs752107 in Wnt/β-catenin signaling pathway strongly associated with the incidence of EH, HF, and IS. As summarized in forest plot (Figure 2), compared with *WNT3A* rs752107 C allele, the T allele conferred an increased risk for EH, HF, and IS.

**Figure 3:**
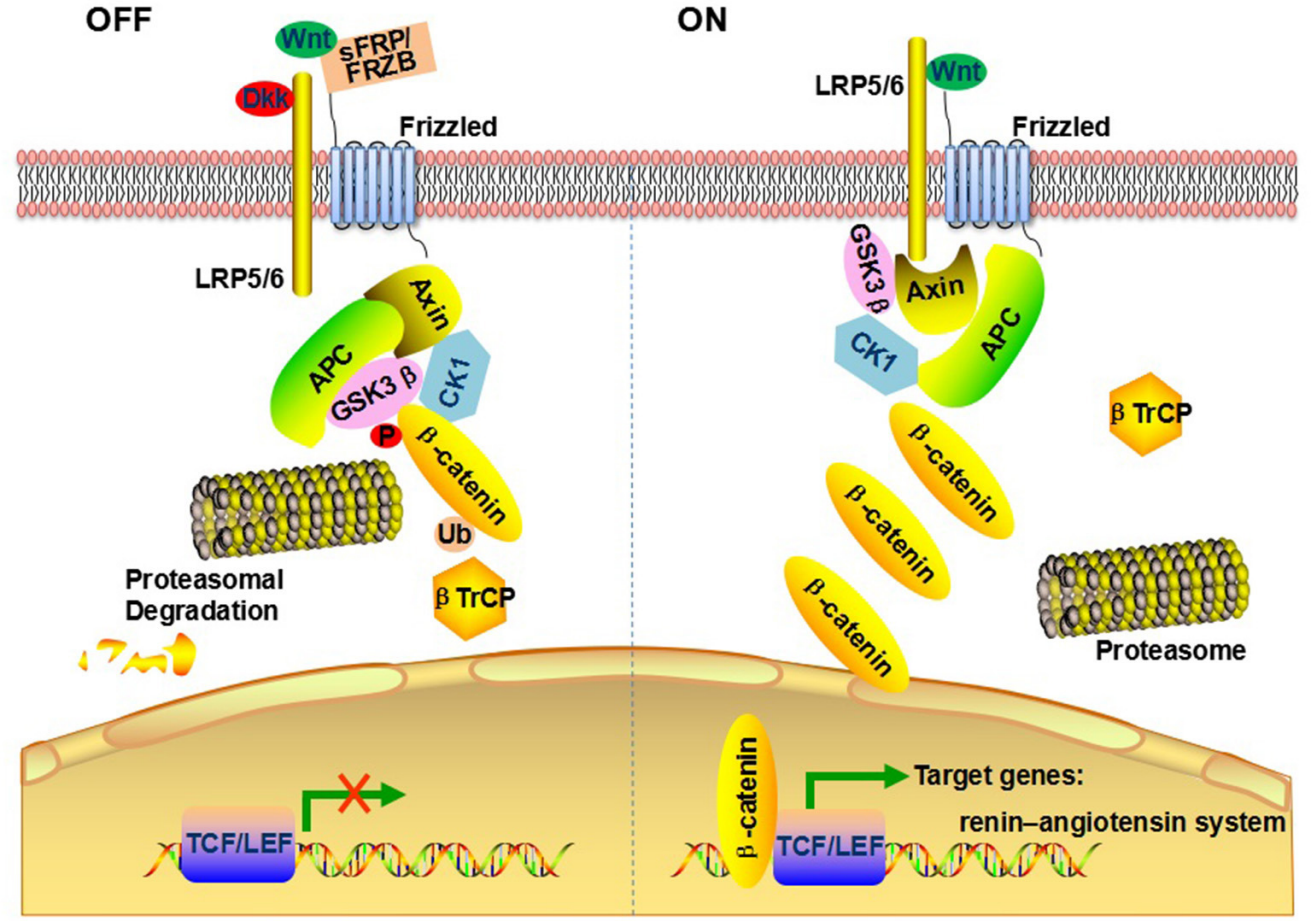
Schematic summary of Wnt/β-catenin signaling pathway.

The canonical Wnt/β-catenin pathway involves the transcriptional co-activator β-catenin, as showed in Figure 3. Transmembrane frizzled (FZD) and low-density lipoprotein-related receptor (LRP) act as co-receptor complexes. In the presence of Wnt, FZD, and LRP complexes are triggered resulting in β-catenin accumulation and translocation to the nucleus. In the nucleus, β-catenin binds to T cell factor (TCF)/lymphoid-enhancer binding factor (LEF), activating transcription of Wnt target genes (16). Wnt/β-catenin signaling can be regulated at many different levels. The expression of *CTNNB1*, the gene encoding β-catenin, could regulate Wnt signaling (17). β-catenin can be destroyed directly by destruction complex in the cytoplasm consisting of axin, adenomatosis polyposis coli (APC), and glycogen synthase kinase3β (GSK3β) (10). The Wnt/β-catenin signaling antagonist includes dickkopf (Dkk)-an endogenous inhibitor of LRP (18), frizzled- related protein (FRZB)- a secreted Wnt antagonist (19), secreted frizzled- related proteins (SFRP)- a competitive antagonist of Wnt (20).

A rapidly emerging field involving the role of Wnt/β-catenin signaling pathway in cardiovascular development and dysfunction has recently drawn extensive attention. Indisputably, pathway mutations of Wnt/β-catenin Signaling pathway are factitive to multiple pathologies of cardiovascular disease. As the common risk factor for CVD, hypertension has been focused by us for a long time (21–23). To define whether the genetic variants of Wnt/β-catenin signaling pathway correlated with hypertension, we conducted case–control studies and screened a total of 95 potentially functional variants within 12 Wnt/β-catenin signaling genes (*WNT3A, WNT3, WNT4, DKK1, DKK2, LRP5, LRP6, CTNNB1, APC, FZD1, FRZB, SFRP1*) mentioned above (Supplementary Table 1). In the exploration cohort of 334 individuals (199 patients with EH and 135 healthy subjects), after Bonferroni correction, we preliminarily validated a genetic association of *WNT3A* rs752107 with EH susceptibility. Then, this genetic association was further confirmed in an enlarged validation cohort of 904 individuals (440 patients with EH and 464 normal control subjects). Interactions between Wnt/β-catenin pathway and renin-angiotensin system (RAS), which plays an essential role in the maintenance of blood pressure homeostasis, may be an interesting way to better understand the function of Wnt/β-catenin pathway during hypertension. Wnt/β-catenin pathway and RAS regulate positively each other during hypertension (24, 25). Zhou et al. demonstrated that Wnt/β-catenin signaling is a master regulator controlling multiple RAS genes, such as *AGT, Renin, ACE, AT1, AT2* (26).

Wnt/β-catenin signaling also get involved in abnormal cardiac remodeling in heart failure (27, 28). In the mouse model of cardiac hypertrophy and heart failure, the cardiac lesions were accompanied with upregulation of multiple Wnt ligands and activation of β-catenin and RAS (29). Malekar et al. found that activation of Wnt signaling is critical and sufficient for maladaptive myocardial hypertrophy and cardiomyopathy (30). Data showed that inhibition of glycogen synthase kinase 3 beta(GSK-3β) during heart failure is protective (31). Besides, a multiancestry GWAS meta-analysis identified a SNP in *WNT2B* (rs12037987) as the novel stroke risk loci (32). Therefore, we then explored the association of *WNT3A* rs752107 with the risk of HF and IS. Interestingly, the results suggest strong links between *WNT3A* rs752107 and the risk of HF and IS in line with expectation.

Wnt3a, as the ligands of Wnt signaling, plays a direct role in the activation of Wnt/β-catenin signaling pathway. Functional mutations in *WNT3A* gene may affect the pathogenesis of cardio-cerebrovascular diseases. *WNT3A* rs752107 polymorphism was identified to be associated with cleft palate and bone mineral density variation (33, 34). The rs752107 polymorphism is located in 3'untranslated regions (3'-UTRs) involved in gene expression control by binding to MicroRNAs (miRNAs) (15). Based on publicly available data, the presence of C allele predicted a stronger binding site for has-miR-892b resulting in a decreased level of *WNT3A* gene expression. Then, eQTL analysis validated that rs752107 C allele is significantly associated with reduced *WNT3A* expression in brain (Table 3). Since Wnt/β-catenin pathway and RAS regulate positively each other during hypertension, the decreased *WNT3A* expression may lead to inactivation of RAS which has a major role in the pathophysiology of hypertension. Otherwise, previous research indicated the inhibition of Wnt signaling could attenuate Wnt3a-induced central blood pressure regulation by downregulating GSK-3β pathway (35).

Limitations of the present study should also be considered. The first limitation is generalizability, while we examined only individuals with Chinese ancestry and results may not be generalizable to other ethnic groups. Additionally, *WNT3A* rs752107 was just predicted to disrupt a miRNA-binding site by web-based tools while lacking experimental validation. Further functional studies are needed to determine the exact implications of this polymorphism.

In summary, we took a pathway-based candidate gene analysis approach to verify the relationship between Wnt/β-catenin signaling and hypertension and related CVDs. And our data highlighted that *WNT3A* rs752107(C > T) was associated with incidence of EH and related CVDs for the first time. However, more representative and comprehensive studies in people of different cohorts and ethnic groups should be considered in the near future. In addition, it will need more *in vitro* and *in vivo* experimental validation to reveal the role of *WNT3A* rs752107 in the function of Wnt3a and Wnt/β-catenin signaling.

## Data Availability Statement

The raw data supporting the conclusions of this article will be made available by the authors, without undue reservation.

## Ethics Statement

The studies involving human participants were reviewed and approved by Medical Ethics Committee of Xiangya Hospital, Central South University. The patients/participants provided their written informed consent to participate in this study. Written informed consent was obtained from the individual(s) for the publication of any potentially identifiable images or data included in this article.

## Author Contributions

X-PC, H-HZ, W-HH, and WZ conceptualized and designed the study protocol. FO and LC collected clinical data and blood samples. HR and J-QL analyzed the data and prepared the manuscript. All authors helped in reviewing the manuscript.

## Conflict of Interest

The authors declare that the research was conducted in the absence of any commercial or financial relationships that could be construed as a potential conflict of interest.
