# Supplementary material for: WNT3A rs752107(C > T) Polymorphism Is Associated With an Increased Risk of Essential Hypertension and Related Cardiovascular Diseases
Source: Front Cardiovasc Med. 2021 Jul 12;8:675222. doi: 10.3389/fcvm.2021.675222 (PMC8310949; doi:10.3389/fcvm.2021.675222)
Supplement: Supplementary file 1 [file Data_Sheet_1.ZIP › Supplementary Table 1.docx]

Table S1 Information of Candidate Genes and SNPs Characteristics.

| SNPs | Gene | Chro:Position | Location | Allele | SNPs | Gene | Chro:Position | Location | Allele |
| --- | --- | --- | --- | --- | --- | --- | --- | --- | --- |
| rs10737462 | *WNT4* | 1:22118482 | 3'UTR | C/T | rs17302049 | *LRP6* | 12:12212391 | intron | A/G |
| rs3765351 | *WNT4* | 1:22119498 | 3'UTR | A/G | rs11054731 | *LRP6* | 12:12223531 | 5'UTR | A/G |
| rs2072920 | *WNT4* | 1:22119772 | 3'UTR | C/T | rs11054738 | *LRP6* | 12:12235093 | intron | A/T |
| rs10917157 | *WNT4* | 1:22126831 | intron | C/T | rs7134001 | *LRP6* | 12:12239425 | intron | C/T |
| rs10917158 | *WNT4* | 1:22127880 | intron | C/G | rs7302808 | *LRP6* | 12:12267298 | 5'UTR | C/T |
| rs2865175 | *WNT4* | 1:22136599 | intron | C/T | rs7136380 | *LRP6* | 12:12267301 | 5'UTR | C/T |
| rs12135916 | *WNT4* | 1:22143622 | 5'UTR | A/G | rs7304561 | *LRP6* | 12:12269471 | 5'UTR | A/G |
| rs56318008 | *WNT4* | 1:22143914 | 5'UTR | C/T | rs7136900 | *LRP6* | 12:12270159 | 5'UTR | A/G |
| rs12038516 | *WNT4* | 1:22144389 | 5'UTR | C/T | rs11658976 | *WNT3* | 17:46789439 | intron | A/G |
| rs7521902 | *WNT4* | 1:22164231 | 5'UTR | A/C | rs4666865 | *FRZB* | 2:182833364 | 3'UTR | A/G |
| rs6426749 | *WNT4* | 1:22384980 | 5'UTR | C/G | rs36090522 | *FRZB* | 2:182867040 | 5'UTR | -/G |
| rs708113 | *WNT3A* | 1:228005052 | 5'UTR | A/T | rs4293535 | *FRZB* | 2:182867244 | 5'UTR | A/T |
| rs13373831 | *WNT3A* | 1:228006090 | 5'UTR | A/G | rs9288087 | *FRZB* | 2:182867309 | 5'UTR | A/G |
| rs964941 | *WNT3A* | 1:228006156 | 3'UTR | C/T | rs12469777 | *FRZB* | 2:182867636 | 5'UTR | C/T |
| rs6672559 | *WNT3A* | 1:228016625 | intron | A/G | rs55840513 | *CTNNB1* | 3:41197554 | 5'UTR | A/C |
| rs1636195 | *WNT3A* | 1:228023888 | intron | A/G | rs60637347 | *CTNNB1* | 3:41197887 | 5'UTR | -/G |
| rs708122 | *WNT3A* | 1:228029296 | intron | G/T | rs3864004 | *CTNNB1* | 3:41198686 | 5'UTR | A/G |
| rs6672422 | *WNT3A* | 1:228029660 | intron | C/T | rs1798802 | *CTNNB1* | 3:41220488 | intron | A/G |
| rs10916258 | *WNT3A* | 1:228032181 | intron | A/C | rs3774369 | *CTNNB1* | 3:41226034 | intron | A/G |
| rs1745423 | *WNT3A* | 1:228036452 | intron | A/C | rs11564459 | *CTNNB1* | 3:41234916 | intron | A/G |
| rs3121310 | *WNT3A* | 1:228037123 | intron | A/C | rs4135385 | *CTNNB1* | 3:41237949 | intron | A/G |
| rs1034792 | *WNT3A* | 1:228041366 | intron | G/T | rs11564475 | *CTNNB1* | 3:41238542 | intron | A/G |
| rs11584499 | *WNT3A* | 1:228048108 | intron | A/G | rs2293303 | *CTNNB1* | 3:41239336 | intron | C/T |
| rs6675092 | *WNT3A* | 1:228052819 | intron | G/T | rs2953 | *CTNNB1* | 3:41239897 | 3'UTR | G/T |
| rs752107 | *WNT3A* | 1:228059650 | 3'UTR | T/C | rs419558 | *DKK2* | 4:106922935 | intron | C/T |
| rs41270175 | *WNT3A* | 1:228060660 | 3'UTR | A/C | rs419764 | *DKK2* | 4:106923000 | 3'UTR | C/T |
| rs41270177 | *WNT3A* | 1:228060807 | 3'UTR | A/G | rs17037102 | *DKK2* | 4:106924637 | missense | A/G |
| rs1896368 | *DKK1* | 10:52309144 | 5'UTR | A/G | rs3733634 | *DKK2* | 4:107036728 | 5'UTR | G/T |
| rs2241529 | *DKK1* | 10:52314997 | synonymous | A/G | rs2704335 | *DKK2* | 4:107037219 | 5'UTR | A/C |
| rs2288335 | *DKK1* | 10:52319294 | intron | A/G | rs6867376 | *APC* | 5:112736773 | intron | C/G |
| rs1569199 | *DKK1* | 10:52319956 | intron | C/T | rs11950612 | *APC* | 5:112751177 | intron | C/T |
| rs1528873 | *DKK1* | 10:52342245 | intron | G/T | rs2439591 | *APC* | 5:112753783 | intron | C/T |
| rs1881747 | *DKK1* | 10:52573815 | intron | C/T | rs1816769 | *APC* | 5:112774082 | intron | C/G |
| rs312009 | *LRP5* | 11:68309770 | intron | C/T | rs2289485 | *APC* | 5:112781032 | intron | G/T |
| rs682429 | *LRP5* | 11:68311851 | intron | A/G | rs2289484 | *APC* | 5:112781076 | intron | C/T |
| rs4988300 | *LRP5* | 11:68321363 | intron | G/T | rs501250 | *APC* | 5:112820096 | intron | C/G |
| rs312778 | *LRP5* | 11:68340864 | intron | C/T | rs201988789 | *APC* | 5:112841339 | missense | C/G |
| rs638051 | *LRP5* | 11:68373946 | intron | A/G | rs397768 | *APC* | 5:112845879 | 3'UTR | A/G |
| rs2306862 | *LRP5* | 11:68410042 | synonymous | C/T | rs565453 | *APC* | 5:112849696 | 3'UTR | A/C |
| rs3736228 | *LRP5* | 11:68433827 | missense | C/T | rs481789 | *APC* | 5:112850367 | 3'UTR | A/C |
| rs7316466 | *LRP6* | 12:12115592 | 3'UTR | C/T | rs2232158 | *FZD1* | 7:91264787 | 5'UTR | C/G |
| rs2284396 | *LRP6* | 12:12122001 | intron | C/T | rs3750145 | *FZD1* | 7:91267516 | 3'UTR | A/G |
| rs11054701 | *LRP6* | 12:12136222 | intron | C/T | rs1052015 | *FZD1* | 7:91268416 | 3'UTR | A/C |
| rs2302685 | *LRP6* | 12:12148964 | missense | C/T | rs4736958 | *SFRP1* | 8:41261978 | 3'UTR | C/T |
| rs7305037 | *LRP6* | 12:12175830 | intron | C/T | rs12914 | *SFRP1* | 8:41262633 | 3'UTR | A/G |
| rs7966410 | *LRP6* | 12:12184940 | intron | C/G | rs1127379 | *SFRP1* | 8:41263761 | 3'UTR | A/G |
| rs10772539 | *LRP6* | 12:12186690 | intron | G/T | rs10088390 | *SFRP1* | 8:41264249 | 3'UTR | C/G |
| rs1181332 | *LRP6* | 12:12208985 | intron | A/G |  |  |  |  |  |
