# Supplementary material for: WNT3A rs752107(C > T) Polymorphism Is Associated With an Increased Risk of Essential Hypertension and Related Cardiovascular Diseases
Source: Front Cardiovasc Med. 2021 Jul 12;8:675222. doi: 10.3389/fcvm.2021.675222 (PMC8310949; doi:10.3389/fcvm.2021.675222)
Supplement: Supplementary file 1 [file Data_Sheet_1.ZIP › Supplementary Table 2.docx]

Table S2 Baseline Characteristics of participants in the enlarged validation cohort and HF, IS cohorts

| Characteristics | Controls | Cases | | |
| --- | --- | --- | --- | --- |
|  |  | EH | HF | IS |
| Gender (male:female) | 51.2:48.8 | 54.3:45.7 | 56.1:43.9 | 53.8:46.2 |
| Age (years) | 58±7 | 60±6 | 59±8 | 61±9 |
| Smoking(%) | 17.0 | 20.1 | 18.9 | 19.6 |
| Drinking(%) | 18.5 | 24.6 | 22.2 | 22.8 |
| SBP (mmHg) | 118±13 | 165±18 | 132±25 | 147±22 |
| DBP (mmHg) | 75±9 | 93±11 | 80±16 | 87±13 |
| FBG (mmol/L) | 5.03±1.21 | 5.66±1.89 | 5.53±1.53 | 5.72±1.92 |
| TG(mmol/L) | 1.81±1.53 | 1.50±1.07 | 1.43±1.23 | 1.85±1.31 |
| TC(mmol/L) | 4.91±1.04 | 4.81±0.97 | 4.15±1.03 | 4.66±1.23 |
| HDLC(mmol/L) | 1.55±0.59 | 2.10±0.52 | 1.10±0.28 | 1.29±0.37 |
| LDLC(mmol/L) | 2.51±0.79 | 2.28±0.69 | 2.40±0.80 | 2.73±0.88 |
| Cr(umol/L) | 69.25±18.37 | 67.00±26.56 | 89.76±37.12 | 83.60±22.91 |

Abbreviations: SBP, systolic blood pressure; DBP, diastolic blood pressure; FBG, fasting blood glucose; TG, triglyceride; TC, total cholesterol; HDLC, high-density lipoproteincholesterol; LDLC, low-density lipoproteincholesterol; Cr, creatinine. Values were expressed as mean ± SD.
