# Supplementary material for: WNT3A rs752107(C > T) Polymorphism Is Associated With an Increased Risk of Essential Hypertension and Related Cardiovascular Diseases
Source: Front Cardiovasc Med. 2021 Jul 12;8:675222. doi: 10.3389/fcvm.2021.675222 (PMC8310949; doi:10.3389/fcvm.2021.675222)
Supplement: Supplementary file 1 [file Data_Sheet_1.ZIP › Supplementary Table3.docx]

Table S3. The valid SNPs included in the statistics

| SNP | Gene | F_A | F_U | CHISQ | *P*-value | OR | L95 | U95 |
| --- | --- | --- | --- | --- | --- | --- | --- | --- |
| rs10737462 | *WNT4* | 0.270 | 0.293 | 0.405 | 0.525 | 0.89 | 0.63 | 1.27 |
| rs3765351 | *WNT4* | 0.159 | 0.173 | 0.232 | 0.630 | 0.90 | 0.59 | 1.38 |
| rs2072920 | *WNT4* | 0.111 | 0.119 | 0.078 | 0.781 | 0.93 | 0.57 | 1.53 |
| rs10917157 | *WNT4* | 0.259 | 0.256 | 0.010 | 0.920 | 1.02 | 0.71 | 1.46 |
| rs10917158 | *WNT4* | 0.108 | 0.119 | 0.190 | 0.663 | 0.90 | 0.55 | 1.47 |
| rs2865175 | *WNT4* | 0.040 | 0.048 | 0.244 | 0.622 | 0.83 | 0.39 | 1.77 |
| rs12135916 | *WNT4* | 0.188 | 0.200 | 0.144 | 0.705 | 0.93 | 0.62 | 1.38 |
| rs56318008 | *WNT4* | 0.514 | 0.459 | 1.865 | 0.172 | 1.25 | 0.91 | 1.71 |
| rs12038516 | *WNT4* | 0.243 | 0.250 | 0.038 | 0.846 | 0.96 | 0.67 | 1.39 |
| rs7521902 | *WNT4* | 0.475 | 0.496 | 0.270 | 0.604 | 0.92 | 0.67 | 1.26 |
| rs6426749 | *WNT4* | 0.286 | 0.230 | 2.675 | 0.102 | 1.35 | 0.94 | 1.93 |
| rs708113 | *WNT3A* | 0.372 | 0.313 | 2.335 | 0.127 | 1.30 | 0.93 | 1.81 |
| rs13373831 | *WNT3A* | 0.251 | 0.189 | 3.553 | 0.059 | 1.44 | 0.98 | 2.11 |
| rs6672559 | *WNT3A* | 0.312 | 0.316 | 0.009 | 0.923 | 0.98 | 0.70 | 1.38 |
| rs1636195 | *WNT3A* | 0.097 | 0.034 | 9.516 | 0.002 | 3.08 | 1.46 | 6.52 |
| rs708122 | *WNT3A* | 0.317 | 0.371 | 2.008 | 0.156 | 0.79 | 0.56 | 1.10 |
| rs6672422 | *WNT3A* | 0.122 | 0.138 | 0.375 | 0.540 | 0.86 | 0.54 | 1.38 |
| rs10916258 | *WNT3A* | 0.193 | 0.215 | 0.457 | 0.499 | 0.88 | 0.60 | 1.29 |
| rs1745423 | *WNT3A* | 0.486 | 0.399 | 4.823 | 0.028 | 1.43 | 1.04 | 1.97 |
| rs3121310 | *WNT3A* | 0.420 | 0.330 | 5.399 | 0.020 | 1.47 | 1.06 | 2.04 |
| rs1034792 | *WNT3A* | 0.212 | 0.250 | 1.287 | 0.257 | 0.80 | 0.55 | 1.17 |
| rs11584499 | *WNT3A* | 0.174 | 0.211 | 1.422 | 0.233 | 0.79 | 0.53 | 1.17 |
| rs6675092 | *WNT3A* | 0.116 | 0.085 | 1.625 | 0.202 | 1.41 | 0.83 | 2.41 |
| rs752107 | *WNT3A* | 0.296 | 0.168 | 13.640 | 0.000 | 2.08 | 1.40 | 3.08 |
| rs41270175 | *WNT3A* | 0.171 | 0.205 | 1.188 | 0.276 | 0.80 | 0.54 | 1.20 |
| rs41270177 | *WNT3A* | 0.207 | 0.250 | 1.668 | 0.197 | 0.78 | 0.54 | 1.14 |
| rs1896368 | *DKK1* | 0.363 | 0.374 | 0.082 | 0.774 | 0.95 | 0.69 | 1.32 |
| rs2241529 | *DKK1* | 0.312 | 0.258 | 2.242 | 0.134 | 1.31 | 0.92 | 1.86 |
| rs2288335 | *DKK1* | 0.069 | 0.049 | 1.112 | 0.292 | 1.45 | 0.73 | 2.88 |
| rs1569199 | *DKK1* | 0.240 | 0.189 | 2.317 | 0.128 | 1.36 | 0.92 | 2.01 |
| rs1528873 | *DKK1* | 0.230 | 0.187 | 1.760 | 0.185 | 1.30 | 0.88 | 1.92 |
| rs1881747 | *DKK1* | 0.065 | 0.107 | 3.800 | 0.051 | 0.57 | 0.33 | 1.01 |
| rs682429 | *LRP5* | 0.429 | 0.439 | 0.062 | 0.804 | 0.96 | 0.70 | 1.32 |
| rs4988300 | *LRP5* | 0.267 | 0.302 | 0.975 | 0.323 | 0.84 | 0.59 | 1.19 |
| rs312778 | *LRP5* | 0.104 | 0.137 | 1.617 | 0.204 | 0.73 | 0.45 | 1.19 |
| rs638051 | *LRP5* | 0.318 | 0.358 | 1.151 | 0.283 | 0.84 | 0.60 | 1.16 |
| rs2306862 | *LRP5* | 0.190 | 0.239 | 2.306 | 0.129 | 0.75 | 0.51 | 1.09 |
| rs3736228 | *LRP5* | 0.196 | 0.254 | 2.998 | 0.083 | 0.72 | 0.49 | 1.05 |
| rs7316466 | *LRP6* | 0.141 | 0.174 | 1.276 | 0.259 | 0.78 | 0.51 | 1.20 |
| rs2284396 | *LRP6* | 0.164 | 0.169 | 0.030 | 0.862 | 0.96 | 0.63 | 1.47 |
| rs2302685 | *LRP6* | 0.081 | 0.070 | 0.247 | 0.620 | 1.16 | 0.64 | 2.13 |
| rs7305037 | *LRP6* | 0.282 | 0.276 | 0.029 | 0.865 | 1.03 | 0.72 | 1.47 |
| rs7966410 | *LRP6* | 0.226 | 0.233 | 0.037 | 0.848 | 0.96 | 0.66 | 1.41 |
| rs1181332 | *LRP6* | 0.069 | 0.056 | 0.438 | 0.508 | 1.25 | 0.64 | 2.44 |
| rs17302049 | *LRP6* | 0.199 | 0.169 | 0.908 | 0.341 | 1.22 | 0.81 | 1.85 |
| rs11054731 | *LRP6* | 0.256 | 0.280 | 0.455 | 0.500 | 0.88 | 0.62 | 1.27 |
| rs11054738 | *LRP6* | 0.065 | 0.052 | 0.419 | 0.517 | 1.26 | 0.62 | 2.55 |
| rs7134001 | *LRP6* | 0.155 | 0.172 | 0.297 | 0.586 | 0.89 | 0.58 | 1.36 |
| rs7302808 | *LRP6* | 0.322 | 0.313 | 0.057 | 0.811 | 1.04 | 0.74 | 1.46 |
| rs7136380 | *LRP6* | 0.190 | 0.181 | 0.077 | 0.781 | 1.06 | 0.70 | 1.60 |
| rs7136900 | *LRP6* | 0.091 | 0.082 | 0.159 | 0.690 | 1.12 | 0.64 | 1.97 |
| rs11658976 | *WNT3* | 0.397 | 0.452 | 1.906 | 0.167 | 0.80 | 0.59 | 1.10 |
| rs4666865 | *FRZB* | 0.207 | 0.130 | 6.621 | 0.010 | 1.76 | 1.14 | 2.70 |
| rs4293535 | *FRZB* | 0.447 | 0.479 | 0.338 | 0.561 | 0.88 | 0.57 | 1.36 |
| rs9288087 | *FRZB* | 0.391 | 0.459 | 3.039 | 0.081 | 0.76 | 0.55 | 1.04 |
| rs12469777 | *FRZB* | 0.481 | 0.458 | 0.316 | 0.574 | 1.10 | 0.80 | 1.50 |
| rs55840513 | *CTNNB1* | 0.216 | 0.209 | 0.039 | 0.844 | 1.04 | 0.70 | 1.55 |
| rs3864004 | *CTNNB1* | 0.224 | 0.244 | 0.354 | 0.552 | 0.89 | 0.62 | 1.29 |
| rs1798802 | *CTNNB1* | 0.273 | 0.265 | 0.051 | 0.821 | 1.04 | 0.73 | 1.49 |
| rs3774369 | *CTNNB1* | 0.218 | 0.212 | 0.034 | 0.854 | 1.04 | 0.70 | 1.53 |
| rs11564459 | *CTNNB1* | 0.030 | 0.019 | 0.805 | 0.370 | 1.62 | 0.56 | 4.73 |
| rs4135385 | *CTNNB1* | 0.500 | 0.463 | 0.822 | 0.365 | 1.16 | 0.84 | 1.60 |
| rs11564475 | *CTNNB1* | 0.110 | 0.104 | 0.057 | 0.811 | 1.06 | 0.64 | 1.77 |
| rs2293303 | *CTNNB1* | 0.140 | 0.137 | 0.009 | 0.925 | 1.02 | 0.65 | 1.60 |
| rs2953 | *CTNNB1* | 0.238 | 0.258 | 0.295 | 0.587 | 0.90 | 0.62 | 1.31 |
| rs419558 | *DKK2* | 0.238 | 0.265 | 0.606 | 0.436 | 0.87 | 0.60 | 1.24 |
| rs419764 | *DKK2* | 0.187 | 0.263 | 5.428 | 0.020 | 0.64 | 0.44 | 0.93 |
| rs17037102 | *DKK2* | 0.407 | 0.353 | 1.886 | 0.170 | 1.26 | 0.91 | 1.74 |
| rs3733634 | *DKK2* | 0.263 | 0.263 | 0.000 | 0.997 | 1.00 | 0.70 | 1.43 |
| rs2704335 | *DKK2* | 0.457 | 0.440 | 0.173 | 0.677 | 1.07 | 0.78 | 1.47 |
| rs11950612 | *APC* | 0.180 | 0.152 | 0.851 | 0.356 | 1.23 | 0.79 | 1.91 |
| rs2439591 | *APC* | 0.225 | 0.185 | 1.504 | 0.220 | 1.28 | 0.86 | 1.89 |
| rs1816769 | *APC* | 0.133 | 0.109 | 0.727 | 0.394 | 1.25 | 0.75 | 2.08 |
| rs2289485 | *APC* | 0.119 | 0.089 | 1.506 | 0.220 | 1.39 | 0.82 | 2.34 |
| rs2289484 | *APC* | 0.404 | 0.326 | 4.129 | 0.042 | 1.40 | 1.01 | 1.95 |
| rs501250 | *APC* | 0.121 | 0.075 | 3.739 | 0.053 | 1.71 | 0.99 | 2.96 |
| rs397768 | *APC* | 0.216 | 0.164 | 2.594 | 0.107 | 1.40 | 0.93 | 2.11 |
| rs565453 | *APC* | 0.210 | 0.175 | 1.171 | 0.279 | 1.25 | 0.83 | 1.87 |
| rs481789 | *APC* | 0.315 | 0.202 | 3.698 | 0.054 | 1.81 | 0.98 | 3.34 |
| rs2232158 | *FZD1* | 0.500 | 0.485 | 0.139 | 0.709 | 1.06 | 0.77 | 1.46 |
| rs3750145 | *FZD1* | 0.224 | 0.187 | 1.361 | 0.243 | 1.26 | 0.85 | 1.86 |
| rs1052015 | *FZD1* | 0.203 | 0.184 | 0.332 | 0.565 | 1.13 | 0.75 | 1.71 |
| rs4736958 | *SFRP1* | 0.287 | 0.272 | 0.149 | 0.699 | 1.07 | 0.75 | 1.53 |
| rs12914 | *SFRP1* | 0.058 | 0.113 | 6.266 | 0.012 | 0.49 | 0.27 | 0.86 |
| rs1127379 | *SFRP1* | 0.390 | 0.440 | 1.571 | 0.210 | 0.81 | 0.59 | 1.12 |
| rs10088390 | *SFRP1* | 0.352 | 0.361 | 0.053 | 0.817 | 0.96 | 0.69 | 1.34 |
